# Influence of Wireworm Diet on its Susceptibility to and Control With the Entomopathogenic Fungus *Metarhizium brunneum* (Hypocreales: Clavicipitaceae) in Laboratory and Field Settings

**DOI:** 10.1093/jee/toac198

**Published:** 2022-12-28

**Authors:** Lara Reinbacher, Eva Praprotnik, Jaka Razinger, Sven Bacher, Giselher Grabenweger

**Affiliations:** Agroscope, Extension Arable Crops, Departement Plants and Plant Products, Zurich, Switzerland; University of Fribourg, Department of Biology, Unit of Ecology and Evolution, Fribourg, Switzerland; Research Institute of Organic Agriculture FiBL, Departement Crop Sciences, Frick, Switzerland; Agricultural Institute of Slovenia, Plant Protection Department, Ljubljana, Slovenia; Agricultural Institute of Slovenia, Plant Protection Department, Ljubljana, Slovenia; University of Fribourg, Department of Biology, Unit of Ecology and Evolution, Fribourg, Switzerland; Agroscope, Extension Arable Crops, Departement Plants and Plant Products, Zurich, Switzerland

**Keywords:** *Agriotes*, entomopathogenic fungi, cover crop, biological control, host plant

## Abstract

Entomopathogenic fungi (EPF) represent promising control agents against wireworms but success in field experiments is inconsistent. The physiological condition of the targeted insect is crucial for its ability to withstand fungal infection. In particular, nutritional status is among the most important determinants of the insects’ immune defense. In this study, we investigated the effects of diet on the development of the wireworm *Agriotes obscurus* (L.) (Coleoptera: Elateridae) and its subsequent susceptibility to the fungal pathogen *Metarhizium brunneum* (Petch) (Hypocreales: Clavicipitaceae) in a pot experiment. After being reared on one of five plant diets for eight weeks, wireworms were exposed to an environment inoculated with the EPF and monitored for their susceptibility to fungal infection. We then performed a field experiment in which three plant diets (clover, radish, and a cover crop mix), selected according to the insects’ performance in the laboratory experiment, were grown as a cover crop with EPF application. Plant diet influenced growth and development of larvae, but there were no strong differences in susceptibility toward fungal infection in the laboratory experiment. Damage levels in EPF-treated plots in the field varied depending on the cover crop. Damage was highest in plots planted with a mix of cover crop species, whereas damage was lowest in plots with clover or radish alone. This agrees with the laboratory results where insect performance was inferior when fed on clover or radish. Cover crop effects on wireworm damage in the subsequent cash crop may thus vary depending on the cover crop species selected.

The use of entomopathogenic fungi (EPF) for insect biocontrol represents an environmentally friendly alternative to conventional chemical pesticides (Khan and Ahmad 2019). There are many examples of their successful implementation (de Faria and Wraight 2007), such as the control of spittlebugs (Hemiptera: Cercopidae) in Brazil (Mascarin et al. 2019) or white grubs (Coleoptera: Scarabaeidae) in Switzerland (Keller 1986, 2000). Nevertheless, biological control with EPF remains challenging as its efficacy can be easily disrupted by environmental conditions and this has limited its use (Skinner et al. 2014). In addition to direct effects on EPF efficacy arising from abiotic (e.g., temperature, humidity, UV-radiation) and biotic factors (e.g., competition with other microorganisms (Jaronski 2007)), there are also indirect plant-mediated effects on insect–entomopathogen interactions (Cory and Hoover 2006). These effects have been described both in relation to the fungus, for example, root exudates functioning as a fungal nutrient source (Bruck 2009), and in relation to the insect, with the nutritional value and secondary metabolites of the plant influencing insect performance and immune defense (Simpson and Raubenheimer 2001, Shikano et al. 2017).

Most studies on the influence of nutrition on insect immune defense against EPF have been conducted with the aim of improving basic knowledge of the insect immune system and the multitrophic relationships between insect and entomopathogen. It has been shown that certain plant diets can enhance resistance or tolerance to the pathogen, for example enriching the diet of the corn earworm, *Helicoverpa zea* (Boddie) (Lepidoptera: Noctuidae), with the glycoalkaloid α-tomatine lowers the mortality of larvae infected with the EPF *Metarhizium* (*Nomuraea*) *rileyi* (Farlow) (Hypocreales: Clavicipitaceae) (Gallardo et al. 1990). Dietary choices can even be seen as a form of self-medication (de Roode and Hunter 2019), as seen in locusts that improve their chances of surviving a *Metarhizium acridum* (Driver and Milner) infection after decreasing intake of protein (Graham et al. 2014). However, undernutrition or malnutrition can also lead to increased susceptibility to EPF, such as in nonbloodfed *Anopheles gambiae* (s.s. Giles) (Diptera: Culicidae) (Mnyone et al. 2011), in *Drosophila melanogaster* (Meigen) (Diptera: Drosophilidae) receiving high-dose glucose diets (Unckless et al. 2015), or in codling moth [*Cydia pomonella* (L.) (Lepidoptera: Tortricidae)] larvae deprived of vitamin C (Pristavko and Dovzhenok 1974).

Research on the practical implementation of these interactions for biological control, however, is limited to few species, such as *Bactericera cockerelli* (Sulc.) (Hemiptera: Triozidae) (Ocampo‐Hernández et al. 2019), *Tetranychus urticae* (Koch) (Trombidiformesy: Tetranychidae) (Gatarayiha et al. 2010), *Hyphantria cunae* (Drury) (Lepidoptera: Erebidae) (Zibaee et al. 2013) and *Bemisia tabaci* (Gennadius) (Hemiptera: Aleyrodidae) (Santiago-Álvarez et al. 2006, Olleka et al. 2009, Tian et al. 2016, Zafar et al. 2016). Previous work has mainly focused on explaining discrepancies in the efficacy of EPF and evaluation of their suitability for specific crops. In contrast, in this study we aim to actively integrate potential plant diet effects into the biocontrol system to enhance field efficacy. Here we investigate if wireworm (*Agriotes* spp.) control with EPF can be improved by providing an ill-suited diet to the larvae in the cover crop.

Wireworms, the larvae of click beetles (Coleoptera: Elateridae), cause damage in several important crops such as maize and potato (Vernon and van Herk 2013, Furlan et al. 2017) and there are currently no reliable control options available to farmers (Veres et al. 2020). The EPF *Metarhizium brunneum* (Petch) has been identified as one of the most promising control methods (Ritter and Richter 2013) and several studies in Europe and North America have its potential for wireworm control in the field (e.g., Kabaluk and Ericsson 2007, Brandl et al. 2017, Mayerhofer et al. 2017, Antwi et al. 2018, Sharma et al. 2020). However also here EPF efficacy was often inconsistent and requires improvement.

Due to their long life cycle, wireworms can be found in soil all year round (Traugott et al. 2015). EPF application is thus not necessarily linked to the vulnerable crop but can be executed at any time during the agricultural crop rotation. As generalist herbivores, wireworms feed on a wide array of subterranean plant parts. While they show some preferences for food types like grasses and legumes (Schallhart et al. 2012), they rarely disperse horizontally with estimations ranging from 10–20 cm to a maximum of 1–1.5 m (Dobrovolsky 1970, Schallhart et al. 2011) provided that there is some kind of food source available (Dobrovolsky 1970, Schallhart et al. 2011). These characteristics give us a unique chance to influence the plant food resources available to wireworms during EPF application.

Previous studies have shown that application of the EPF *M. brunneum* to cover crops preceding potato cultivation can reduce wireworm abundance in the field. However, the effect on yield (potato tuber quality) was low (Rogge et al. 2017, Reinbacher et al. 2021). We hypothesized that the suitability of cover crops as a food resource for wireworms varies. Consequently, the choice of cover crop will have an influence on the insects’ immune defense. If the cover crop is an ill-suited food source for wireworms, it should increase susceptibility of the wireworms towards pathogens, and consequently improve the efficacy of EPF treatments.

Several plant species are recommended as cover crops preceding potato production. Since potatoes require large amounts of nitrogen, leguminous crops like clover with their ability to fix nitrogen are particularly useful preceding potato (Canali et al. 2012). Other plant species help to prevent pest problems, e.g., oilseed radish and bristle oat are used as cover crop to prevent plant-parasitic nematodes in potatoes (Schmidt et al. 2017). To test our hypothesis, we used a selection of these recommended cover crops, namely berseem clover, bristle oat, oilseed radish and a cover crop mix, to test the impact of plant diet on wireworm [*Agriotes obscurus* (L.)] development and susceptibility to fungal infection in climate-controlled assays and wireworm damage field trials.

## Materials and Methods

### Effects of Plant Diet on Wireworm Development and Susceptibility to Fungal Infection

**Fig. 1.**
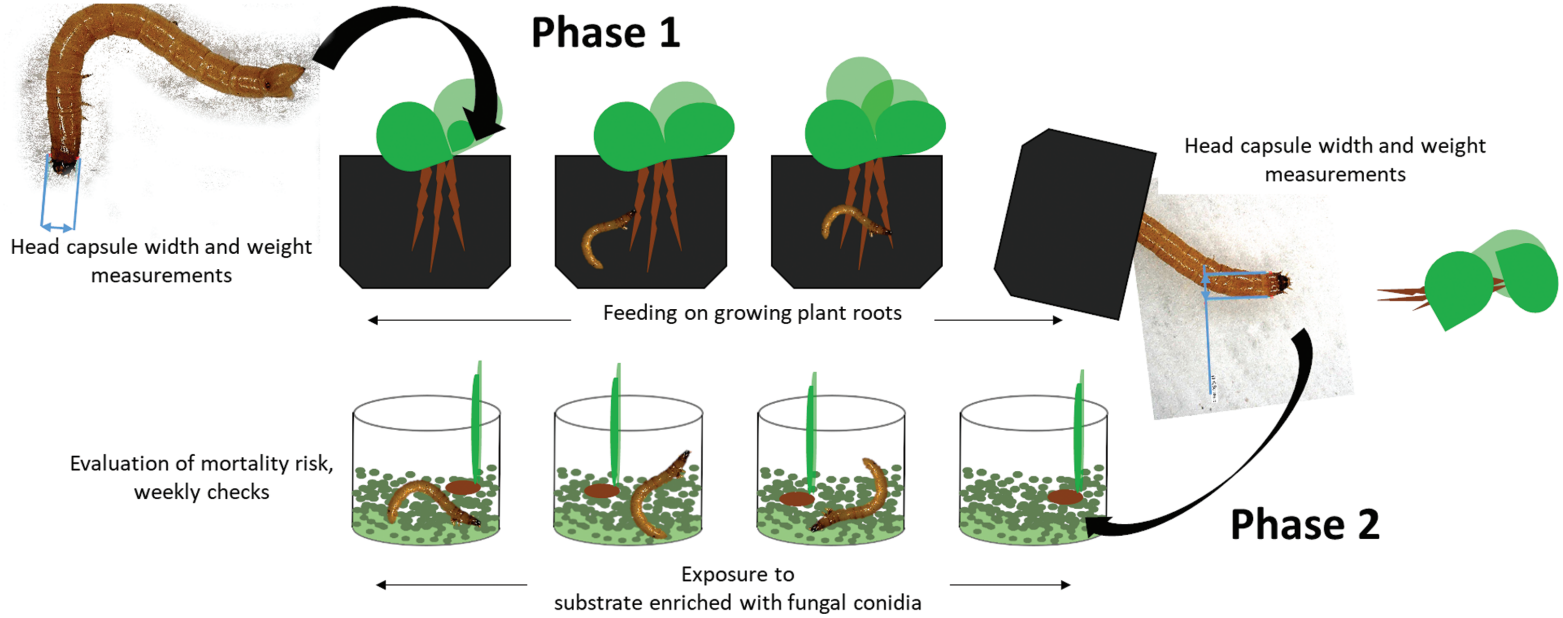
Schematic representation of the two-phase experiment under climate-controlled conditions to evaluate plant diet effects on the susceptibility of wireworms to the EPF *M. brunneum* ART2825.

To evaluate the effect of plant diet on the susceptibility of wireworms to EPF, a two-phase experiment was conducted under climate-controlled conditions. In the first phase, wireworm development was quantified during growth on one of five plant diets in a pot experiment. In the second phase, the same wireworms were used in a susceptibility test in which they were exposed to *Metarhizium* conidia and their survival and mycosis were quantified in the laboratory (see Fig. 1). The whole experiment (Phase 1 and 2) was carried out twice (replicate 1 and 2) with a sample size of *n* = 30 per treatment, summing up to a total of 300 tested wireworms (2 independent replicates * 5 treatments * 30 wireworms per treatment). Experiments were performed under the same conditions with the exception of larval instars used and test duration. The first replicate (Rep 1) of the experiment was started with larval instar 4 and 5, while the second replicate (Rep 2) was started with larval instar 6 (Klausnitzer 1994). According to Sufyan et al. (2014) head widths of *A. obscurus* larval instars show considerable variation which may make accurate distinction between instars difficult. Thus, in the following, we present the head width measurements in mm instead of assigning them to instars. For further information on larval instars see Supplementary Material Fig S1. Test duration of the first phase was eight weeks in both experiments; the second phase of the experiment was eight weeks (Rep 1) or 13 wk (Rep 2).

### Wireworm Rearing

*A. obscurus* larvae originated from a laboratory stock established according to Kölliker et al. (2009). In short, adult elaterid beetles were collected from meadows in June and determined morphologically to species level. A stock was established by placing 40 individuals in a pot with mesh caging containing a grass mixture of *Festuca rubra* (L.) (Poales: Poaceae), *F. pratensis* (Huds.), *Poa pratensis* (L.) (Poales: Poaceae), and *Lolium perenne* (L.) (Poales: Poaceae) and potting soil as oviposition substrate. After death of the adults, the mesh caging was removed. Larvae were kept in the pots for another six months and were then transferred to a cool room (10°C). Larval health was assessed before experiments, based on movement behavior (van Herk and Vernon 2013): only larvae showing normal and spontaneous movement were included in the test.

### Wireworm Development on Plant Diets (Phase 1)

Wireworms were tested on the roots of one of five plant diets, chosen from plants that are frequently used as cover crop preceding potatoes. Three single plant diets consisted of berseem clover [*Trifolium alexandrinum* (L.) (Fabales: Fabaceae), variety: Tigri], bristle oat [*Avena strigosa* (Schreb.) (Poales: Poaceae), variety: PRATEX], and oilseed radish [*Raphanus sativus* (L.) (Brassicales: Brassicaceae) var. *oleiformis*, variety: Valencia]. In addition, we included one treatment with a cover crop mix, containing bristle oat, berseem clover, ramtil (*Guizotia abyssinica* (Asterales: Asteraceae) (Cass.)) and phacelia (*Phacelia tanacetifolia* (Boraginales: Boraginaceae) (Bentham), variety: Stala) to allow for diet mixing. Diet mixing has often been found to positively influence the performance of generalist insect herbivores (Unsicker et al. 2008), and so wireworms might be expected to perform best on this diet. Finally, potato slices (mean: 15 g, range 1 g) formed the fifth treatment, supplied on the substrate surface as a restricted diet (potato restricted) available for two days every three weeks.

Plants were sown in potting soil before the start of the test. They were transplanted to planting pots (diameter: 11 cm, volume: 0.75 liter) in growth stage BBCH 11 (at first leaf development) and left to grow for a further 10 d. The number of plants per pot was chosen according to the plant density recommendations of the seed producer (Eric Schweizer AG, Thun Switzerland). Wireworms were then randomly assigned to a plant diet and one individual was released in each planting pot. There were 30 pots for each plant type (*n* = 30). The draining holes were sealed with gauze to prevent wireworms from escaping.

To ensure a large enough sample size for the subsequent susceptibility test (phase 2), a single larger planting pot (diameter 30 cm, volume: 14.14 liter) was prepared for each diet with 20 wireworms as a reserve. All pots were arranged randomly in a greenhouse chamber. The plants were grown at 25°C (range: 3°C) and watered every second day. After eight weeks, wireworms were retrieved, weighed and their head capsules measured.

Larval weight and head capsule width were assessed at the beginning of the trial and eight weeks after receiving their assigned diet. Head capsule widths were measured from pictures taken of live wireworms under a digital microscope (VHX 6000, Keyence, Japan). Precision of measurements was 0.001 g for weight and 0.03 mm for head capsule width in five repeated measurements.

### Effect of Plant Diet on Susceptibility to *M. brunneum* (Phase 2)

For the susceptibility test, we used conidia of the entomopathogenic fungus *M. brunneum* isolate ART2825, which is known to cause high mortality rates in *A. obscurus* (Eckard et al. 2014, Rogge et al. 2017). Conidia were taken from fungal cultures maintained on a selective medium (Sabouraud 2% glucose agar (SDA) with the antibiotics cycloheximide (0.05 g liter^−1^), streptomycin sulfate (0.6 g liter^−1^), tetracycline (0.05 g liter^−1^), and the fungicide dodine (50 mg liter^−1^); Strasser et al. 1996). Viability of *M. brunneum* was first confirmed by assessing the percentage of germinated conidia under a microscope. Drops of 50 µl conidia suspension in 0.1% (v/v) aqueous Tween80 (1 × 10^6^ spores ml^−1^) were applied on a solid culture medium suitable for entomopathogenic fungi (Riba and Ravelojoana 1984). After 24 hr incubation (22°C, 70% RH), three samples of 100 conidia were examined under 400× magnification and considered as viable if the germ tube was at least the length of the spore itself. In both repetitions, germination rates were above 95%. Before use in tests, conidia were incorporated into an artificial soil (pH = 6.0 ± 0.5%) containing 74% industrial sand, 20% kaolin clay, 5% peat, and 1% calcium carbonate (OECD, 2016) to a concentration of 1.1 × 10^5^ conidia g^−1^ soil. The concentration of *M. brunneum* conidia in the susceptibility test was adjusted to correspond to the concentration recommended for field application, that is, 10^14^ conidia ha^−1^ (Rogge et al. 2017) at a soil incorporation depth of 6 cm (Reinbacher et al. 2021). To achieve this, we converted conidia per soil surface area into conidia per soil weight using an estimated bulk soil density of 1,500 kg m^−3^ (EFSA 2017). Plastic cups (90 cm^3^) were filled with 30 g of this mixture (i.e., total of 3.3 × 10^6^ conidia per cup) and moistened to 50% of its maximum water holding capacity (ISO 2012). Wireworms were transferred to the cups and continued to be fed with the same diet as in the pots in phase 1. Plant diets were either supplied as pre-germinated seedlings or cut roots. Wireworms that could not be retrieved from the planting pots in phase 1 were replaced with an individual from the reserve from the same diet to obtain initial sample sizes *n* = 30 as in phase 1.

An untreated control (substrate without conidia) was tested in parallel to evaluate the suitability of the artificial soil for wireworms. Wireworms used for this control were not part of the phase 1 experiment and were fed exclusively with potato slices.

All cups were closed with a perforated lid and randomly distributed in plastic boxes (20–24 cups/box). They were stored in darkness at 22°C, 70% RH. Temperature and relative humidity were recorded daily and adjusted if deviating. Water content of the artificial soil was measured weekly by weighing and pots were re-watered if necessary.

To quantify effects of the plant diet on susceptibility to fungal treatment, wireworm mortality was evaluated weekly by visual inspection of movement. When recovered, wireworm cadavers were further incubated under the same conditions and monitored for intersegmental outgrowth of white mycelium and formation of green conidia layers, indicating *Metarhizium* infection.

### Effects of Cover Crop Choice on the Impact of EPF Application on Wireworm Damage in Field Experiments

To assess the effect of different winter cover crops on control of wireworm damage to potatoes using EPF application, on-farm field experiments were conducted in Slovenia (Brinje, 46°05ʹ32.4ʹ N 14°35ʹ55.1ʹ E) and Switzerland (Rüeterswil, 47°15ʹ21.9ʺN 8°59ʹ15.0ʺE). The sites were chosen based on the farmers’ previous experience with wireworm damage and on the occurrence of *Agriotes* spp. wireworms in soil samples. For each plot holes (0.25 m^2^, 40 cm deep) were dug and soil was examined for wireworms. We applied the EPF *M. brunneum* ART2825 together with the sowing of the cover crops in August 2019, quantified the establishment and development of fungal abundance in the soil over the winter and quantified wireworm damage to the subsequent potato crop.

### *M. brunneum* Production in Bulk

The isolate *M. brunneum* ART2825 originates from an infected *A. obscurus* larva from the rearing facility of Agroscope, Switzerland in 2008 (Kölliker et al. 2011). Conidia were harvested from *A. obscurus* cadavers in the Agroscope stock culture to produce single-spore isolates on a selective medium for entomopathogenic fungi (Strasser et al. 1996). Single-spore isolates were used as starting material for mass production of fungus-colonized barley kernels (FCBKs). Conidia were collected by rinsing Petri-dishes with 0.1% (v/v) aqueous Tween80. The fungus was propagated in a sterilized liquid medium (aqueous solution with 3% sucrose, 2.5% yeast extract, 1% peptone, 1% barley flour) incubated at 28°C for five days. Malting barley kernels were boiled for 30 min and autoclaved before inoculation with the liquid culture. FCBKs were produced at 22°C in a solid-state fermenter developed by the Zurich University of Applied Sciences (ZHAW), consisting of a horizontal column reactor (diameter = 50 cm), an air inlet, and a CO_2_ outlet. FCBK were stored at 4°C until application in the field. The number of conidia per gram FCBKs (fresh weight) was determined by first shaking samples on a vortex for 1 min at 1,250 rpm in 0.1% (v/v) aqueous Tween80 and subsequently counting conidia densities in the supernatant with a haemocytometer. Conidia viability was confirmed within the week of application as described above in Section Effect of Plant Diet on Susceptibility to *M. brunneum* (Phase 2). In all FCBK used, germination rates exceeded 95%.

### Experimental Design and Application Procedure

Experimental winter cover crops consisted of berseem clover and oilseed radish, which were shown to be ill-suited as a nutritional resource for wireworms in the laboratory experiment (see below), and the cover crop mix, which was shown to be favourable for wireworm performance. Additionally, bare fallow was introduced as a fourth ‘cover crop’ treatment to simulate a restricted diet. Cover crop treatments were combined with an *M. brunneum* soil treatment. *M. brunneum* was applied as FCBKs manually distributed on the plots (3 m width and 9 m length) at a density of 10^14^ conidia ha^−1^ and incorporated into the soil with a rotary harrow (CH) or by rake (SLO) to a depth of 6 cm. Following the application of FCBKs, cover crops were sown by hand at 32 kg ha^−1^ (mix) or 25 kg ha^−1^ (clover and radish). In addition to the EPF treatments, the cover crop mix and the bare fallow treatment were also set up in the field without EPF application. Consequently, field tests consisted of a total of six treatments, which were integrated into a completely randomized design with five replicates per treatment (30 plots per field site in total).

### *Metarhizium* Spp. Abundance in the Soil

The abundance of *Metarhizium* spp. in the soil was determined by counting the number of colony-forming units (CFUs) per gram of soil (Kessler et al. (2003). CFU counts were made before (CFU_0_) and one month after application (CFU_1_) as well as 7 months after application (CFU_7_), shortly before the cover crop was plowed under. We took five randomly located soil samples per plot with a soil core borer (diameter 6 cm, depth 6 cm), pooled the samples and mixed them thoroughly to prepare one, homogenous composite sample per plot. From this substrate, three sub-samples of 20 g were taken and individually suspended in 0.18% Na_4_P_2_O_7_ and dispersed on a selective medium in Petri dishes (Strasser et al. 1996). Petri dishes were incubated in darkness at 22 °C and 70% RH. The water content of each soil sample was measured gravimetrically. After 2 wk, colonies were counted and the number of CFUs per gram of soil (dry weight) was calculated. The median of three Petri dishes from each plot was used for analysis.

### Evaluation of Wireworm Damage

In April 2020, potatoes (Variety Agria (CH) or Savinja (SLO)) were planted on the experimental fields in four rows per plot (distance between rows 0.75 m), following plowing of winter cover crop treatments. Wireworm damage was evaluated at harvest in August 2020 by visual inspection of potato tubers for holes caused by wireworm feeding. Damage was determined according to the European and Mediterranean Plant Protection Organization (EPPO) standards PP1/46 (Anonymous 2005) by sampling 100 tubers per plot. Tubers were taken randomly from the two inner potato rows (BBCH 99; Hack et al. 1993). They were into undamaged tubers (no wireworm feeding holes visible) and damaged (one or more feeding holes visible).

### Statistical Analysis

The statistical software R (version 4.1.0; R Core Team, 2021) was used for all analyses, and package ‘ggplot2’ (version 3.3.3, 2020, (Wickham et al. 2016)) for all figures. Effects of plant diet on larval development of *A. obscurus* were tested with linear models fitted by the Laplace approximation, using the package ‘lme4’ (version 1.1-27, 2021; Bates et al. [2015]), with an increase in weight (mg) or head capsule width (mm) during eight weeks of feeding as the response variable. Fixed effects were plant diet, start weight/head capsule width, and replicate. Multiple comparisons among the plant diets were performed using Tukey contrasts in the package ‘multcomp’ (version 1.4-17, 2021, Hothorn et al. [2008]).

A multivariate cox model was fitted to estimate the proportional hazard ratio for wireworm mortality due to fungal infection depending on the plant diet in the laboratory experiment. For this, the package ‘survival’ (version 3.2.11, 2021,Therneau and Grambsch [2000], Therneau [2020]) was used. Plant diet, origin (wireworm taken from single pots or reserve), and replicate (Rep 1 or 2) were included as fixed effects. The difference in test duration of the two replicates was accounted for in the model through censoring. The proportional hazard assumption of the cox model was tested based on the scaled Schoenfeld residuals. To perform a multiple comparison among means of the plant diet groups, Tukey contrasts were used (package ‘multcomp’ [version 1.4-17, 2021, Hothorn et al. (2008)]).

General linear models were fitted to analyze changes in fungal abundance in the soil. To test for heterogeneities among plots in natural *Metarhizium* abundance in the soil, the CFUs g^−1^ soil before application (CFU_0_) were analyzed with a type 2 ANOVA (package ‘car’ [version 3.0-10, 2020, Fox and Weisberg (2019)]) with terms for location and planned treatment. To analyze the increase of CFUs one month after application (CFU_1_-CFU_0_), the model included FCBK application (yes/no) and location (CH/SLO) as fixed effects. To investigate changes in total fungal abundance over time, the model additionally included the fixed effect sampling time after application (1/7 mo). As a second step, the influence of soil cover was assessed separately for plots with and without FCBK application, as not all soil cover treatments were included in the treatment without FCBK application, i.e., Bare fallow + no FCBK, Mix + no FCBK, Bare fallow + FCBK, Mix + FCBK, Radish + FCBK, Clover + FCBK. Total amounts of CFUs were tested with soil cover and location as fixed factors for each sampling date in a separate model, using a type 2 ANOVA (package ‘car’ [version 3.0-10, 2020, Fox and Weisberg (2019)]). Data transformation was performed when necessary to meet the model assumptions of normality of residuals, using cube transformation for the increase in CFUs and log transformation for the total numbers of CFUs after application.

Damage to potatoes was analyzed with a logistic regression (generalized linear model) with the count of damaged (one or more feeding holes)/undamaged (no feeding holes) potatoes as a dependent variable and the treatment (the combination of soil cover and FCBK application) as fixed effect as well as the location of the field site (Slovenia/Switzerland) (‘lme4’ [version 1.1-27, 2021; Bates et al. (2015)]). A multiple comparison among means of the plant diet groups was performed using the Tukey method (package ‘multcomp’version 1.4-17, 2021, Hothorn et al. [2008]). Assumptions for all linear models (linearity, normality, homoscedasticity) were visually examined.

## Results

### Plant Diet Effects on Wireworm Performance

#### Wireworm Development and Plant Diet

**Fig. 2.**
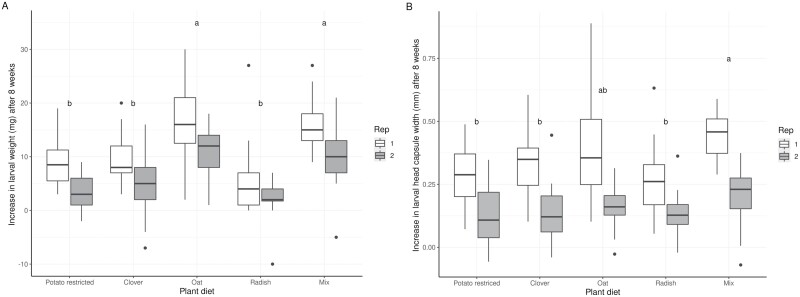
The increase in weight (A) and head capsule width (B) of *A. obscurus* larvae after eight weeks of feeding on the respective plant diets: roots of berseem clover (Clover), cover crop mix (Mix), bristle oat (Oat), or oilseed radish (Radish) (all provided ad libitum), and potato slices renewed every three weeks and available for 2 d (Potato restricted). For each plant diet in both replicates (Rep 1, Rep 2) *n* = 30. Boxplots show the median (central line) and the 25% and 75% quantiles. Different letters indicate significant differences among plant diets at *p* ≤ 0.05 in posthoc Tukey tests.

During eight weeks of feeding, wireworms on average increased in head capsule width (on average 36% of initial width) and weight (on average 127% of initial weight) on all plant diets (Fig. 2). There were statistically significant effects of both plant diet and replicate on the increase of larval weight and head capsule width (Table 1). For weight increase, Tukey posthoc comparison of plant diet groups revealed a significant difference of oats and the mixed diet from all other plant diets. Wireworms feeding on these diets showed a higher weight increase (mix–potato restricted: *t* = 6.46, *p* < 0.001; mix–clover: *t* = 5.42, *p* < 0.001; mix–radish: *t* = 7.05, *p* < 0.001; oat–potato restricted: *t* = 6.54, *p* < 0.001; oat–clover: *t* = 5.54, *p* < 0.001; oat–radish: *t* = 7.61, *p* < 0.001; *n* = 30). In contrast, head capsule widths were only significantly different for the mixed diet in comparison with clover (*t* = 3.65, *p* = 0.003) radish (*z* = 4.06, *p* < 0.001) and potato restricted (*t* = 4.57, *p* < 0.001) (Fig. 2, *n* = 30). In replicate 2 larvae gained less weight (on average 52% of initial weight) compared to replicate 1 (on average 211% of initial weight (*t* = −4.05, *p* < 0.001).

**Table 1.** Results of analysis of variance for the increase in larval weight and head capsule width among wireworms feeding on different plant diets for eight weeks

	Sum of squares	df	*F* value	*p*-value
Source of variation of weight increase				
Plant diet	2609.4	4	25.1	<0.001
Start weight	0.6	1	0.02	0.88
Replicate	534.4	1	20.6	<0.001
Residuals	4878.8	188		
Source of variation of increase in head capsule width				
Plant diet	0.45	4	7.33	<0.001
Start head capsule width	0.002	1	0.14	0.71
Replicate	0.25	1	16.44	<0.001
Residuals	2.88	188		

#### Susceptibility Tests: Wireworm Mortality After Exposure to *M. brunneum*

**Fig. 3.**
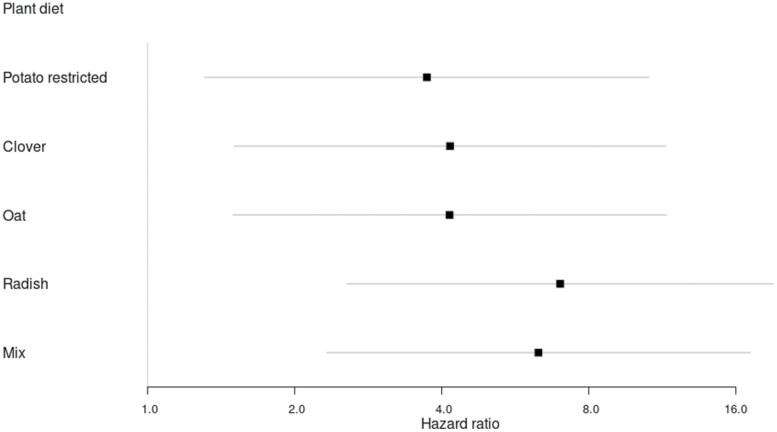
Forest plot showing the effect of plant diet on wireworm mortality due to subsequent treatment with *M. brunneum* ART2825. The hazard ratio (HR), calculated with a multivariate cox model, displays the mortality hazard of the fungus-treated groups in relation to the untreated control. Vertical line (HR = 1, no effect) represents the untreated control. HR > 1 indicates an increase in mortality hazard due to fungal treatment. Black squares indicate the mean hazard ratio, horizontal lines the 95% confidence interval. For each group *n* = 30.

Compared to the untreated control, all fungus-treated groups had a higher hazard of mortality (potato restricted: *z* = 2.46, *p* = 0.01; clover: *z* = 2.75, *p* < 0.01; oat: *z* = 2.73, *p* < 0.01; radish: *z* = 3.8, *p* < 0.001; mix: *z* = 3.62, *p* < 0.001; *n* = 30). Replicate and origin of wireworms (whether from single pots or the reserve pot in phase 1) did not have a significant effect (replicate: *z* = −0.59, *p* = 0.56, origin: *z* = 1.6, *p* = 0.11). Even though multiple comparisons among plant diet means were not statistically significant in the fungus-treated groups, mortality hazard ratios calculated from the model showed tendencies. The radish and mixed plant diets had the highest risk of mortality after EPF exposure (Fig. 3). Cumulative mortalities in the plant diet groups were: untreated control 8.3%, potato restricted 33.3%; clover 38.3%, oat 36.7%; radish 41.7%, and mix 53.3%. In all groups exposed to *M. brunneum,* wireworm cadavers developed *Metarhizium* mycosis (Rep 1 = 84%, Rep 2 = 68%). None of the wireworms dying in the untreated group developed *Metarhizium* mycosis.

### Effects on Wireworm Field Damage

#### Occurrence of *Metarhizium* in Field Soils

**Fig. 4.**
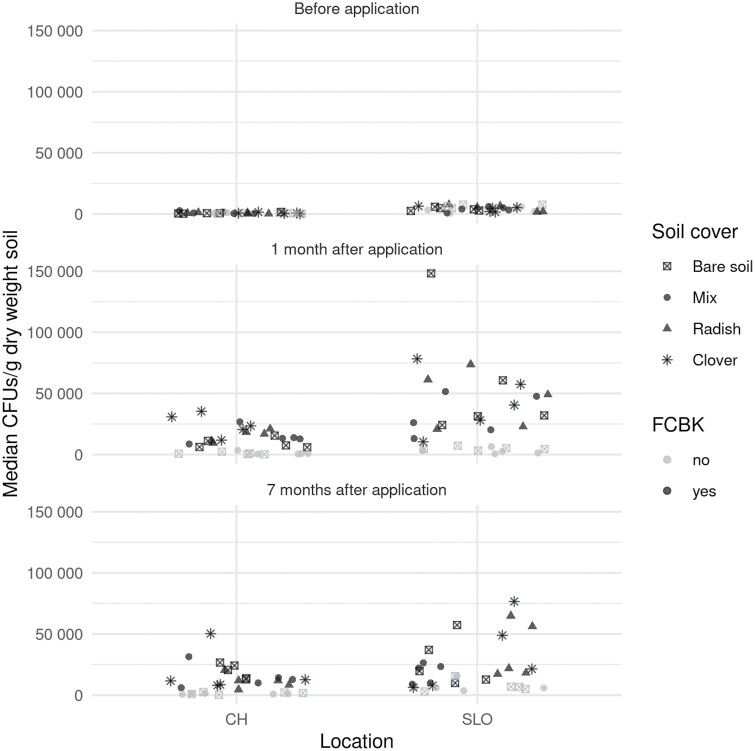
Abundance of *Metarhizium* spp. (CFUs per gram soil dry weight, median of three sub-samples) at the experimental sites in Switzerland (CH) and Slovenia (SLO) on three sampling dates. Plots were treated with *M. brunneum* ART2825 (FCBK yes) or left untreated (FCBK no) and had one of three over-winter soil covers applied (bare fallow, cover crop mix, oilseed radish, berseem clover; *n* = 5).

The abundance of natural *Metarhizium* in soil at the beginning of the trials differed significantly in the two experimental sites (*F* = 82.71, df = 1, *p* < 0.001; *n* = 5). More *Metarhizium* colonies were found at the site in Slovenia, in the range 603–7699 CFUs g^−1^ soil (dry weight), compared to a range of 0–2,697 CFUs g^−1^ soil in Switzerland (Fig. 4). Plots assigned to different treatments did not initially differ in *Metarhizium* abundance (*F* = 0.766, df = 5, *p* = 0.578).

There was a strong increase in the abundance of CFUs one month after application (CFU_1_–CFU_0_) in FCBK-treated plots (*t* = 11.85, *p* < 0.001). Despite the large difference in the natural *Metarhizium* abundance between the sites, the increase in CFUs after application was similar in both locations (*t* = 1.51, *p* = 0.137). *Metarhizium* abundance did not change significantly over the winter (CFU_7_, *t* = −0.046, *p* = 0.963). Soil cover treatment did not have a statistically significant effect on *Metarhizium* abundance in untreated or FCBK-treated plots on either sampling date (untreated: CFU_1_: *F* = 0.59, df = 1, *p* = 0.452; CFU_7_: *F* = 0.01, df = 1, *p* = 0.927; FCBK: CFU_1_: *F* = 0.85, df = 3, *p* = 0.475; CFU_7_: *F* = 0.42, df = 3, *p* = 0.739).

#### Wireworm Damage to Potatoes

**Fig. 5.**
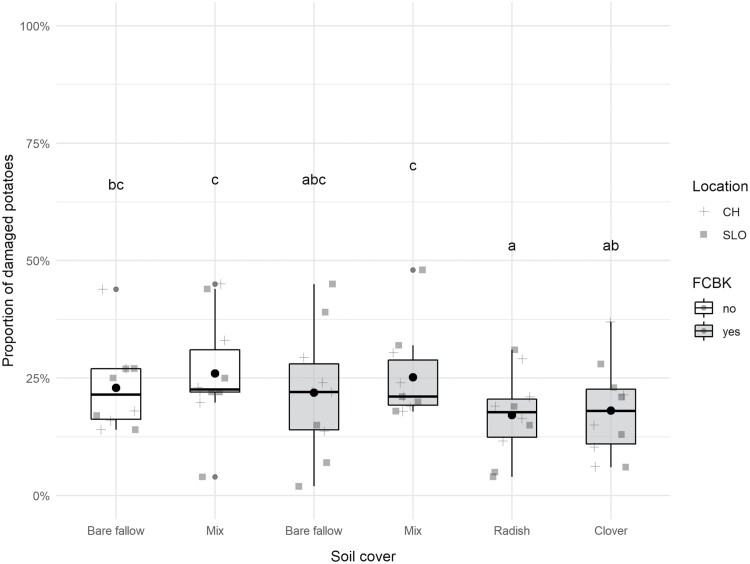
Proportion of wireworm-damaged potatoes (among 100 tubers sampled per plot) in Switzerland (CH) and Slovenia (SLO) in plots treated with *M. brunneum* ART2825 (FCBK yes) and untreated plots (FCBK no) with different soil covers preceding potatoes (bare fallow, cover crop mix, oilseed radish, berseem clover). For each soil cover and treatment combination in the two locations *n* = 5. Boxplots show the median (central line), the 25 and 75% quantile, and the mean (black point). Different letters indicate significant differences among soil cover and treatment combinations at *p* ≤ 0.05 in posthoc Tukey tests.

Wireworm damage was found in all plots at both locations (Fig. 5, *n* = 5). Damage in plots without fungal treatment was similar to plots with (cover crop mix) and without plant cover (bare fallow) (*z* = 1.83, *p* = 0.068). Compared to the bare fallow with no fungal treatment, wireworm damage was significantly reduced in the fungus-treated plots with soil cover radish (*z* = −3.06, *p* = 0.002) and clover (*z* = −2.42, *p* = 0.015) but not in bare fallow (*z* = −0.33, *p* = 0.745) or mix plots (*z* = 1.39, *p* = 0.165).

## Discussion

Results from the laboratory experiment showed that the plant diet influenced wireworm weight gain and development of the head capsule width. We found enhanced weight gain and faster development in wireworm feeding on the cover crop mix than on any single plant diet. While wireworms feeding on oat also gained significantly more weight than other single plant diets, an increase in head capsule width, indicating transitions between larval stages, was similar to the other diets. Food deprivation by time-limited availability of nutritional resources (the potato restricted diet) had similar effects as the ill-suited plant diets. In addition, wireworm performance was different in the two replicates. Older wireworms used in replicate 2 (initially larval instar 6) gained less weight and head capsule width during the same time period than the younger wireworms used in replicate 1 (initially instar 4 and 5). There is evidence from a previous study that the time needed to complete the development of a particular instar increases with larval age (Sufyan et al. 2017). We can conclude that starvation of wireworms, simulated by the potato restricted diet, and exposing wireworms to ill-suited nutritional resources, like radish or clover, have similar negative influences on wireworm performance.

Following our hypothesis, starved wireworms and those fed on ill-suited plant diets should have shown an increased susceptibility to *Metarhizium* infection. This was, however, not the case in our susceptibility assay. Wireworms from all plant diet groups had a similar risk of dying from *Metarhizium* infection. If anything, mortality was slightly (although not significantly) elevated in wireworms feeding on the radish and mixed plant diets, which represent contrasting extremes in terms of suitability as plant diets for wireworms, according to the performance test. Radish was among the ill-suited diets, while the cover crop mix was the best diet, i.e., showing a significant increase in weight and speed of development. Insect responses to multiple stressors such as starvation and pathogen challenges are generally hard to predict. For example, work on the caterpillar *Manduca sexta* (L.) (Lepidoptera: Sphingidae) has shown that food deprivation and low quality food reduce resistance to bacteria but have less effect on resistance to fungi (Adamo et al. 2016). Furthermore, although counterintuitive, facing one stressor can be beneficial for dealing with a second one, since physiological responses may already be activated and are not always specific to one type of stressor (Adamo 2021). Previous studies on wireworms’ risk of infection with entomopathogenic fungi point in the same direction: heavier wireworms show greater mortality from *Metarhizium* infection (Van Herk and Vernon 2011) and the availability of food increases the incidence of *Metarhizium* infections of *A. obscurus* in storage (van Herk et al. 2016). It is, however, not clear if these results were an effect of the plant diet itself, or of other factors such as changes in foraging behavior induced by food availability (see also our field results below).

In conclusion, it appears to be possible to influence wireworm performance negatively by starvation or by exposing them to ill-suited plant diets, but the influence of such measures on immune defense remains unclear. Further studies are needed to clarify whether a diet of ill-suited plants may enhance susceptibility to fungal infection, or whether wireworms are more prone to succumb to pathogens when foraging on optimal nutritional resources. This leads us to the next question, whether imposing ill-suited plants during over-wintering in the field results in higher wireworm susceptibility to *Metarhizium* and lower crop damage.

In our field experiments, six different combinations of soil covers and fungus treatments were put in place. Even though wireworm damages were not highly reduced in any treatment, overall, a statistically significant reduction in damage was observed in fungus-treated radish and clover plots. However, since it was impossible to test all soil covers with and without fungus application, the effect of the fungus application in the set up remains unclear (Fig. 4). *Metarhizium* abundance in the soil was not influenced by the soil cover and plots treated with *M. brunneum* and untreated plots with the same soil cover (cover crop mix or bare fallow) showed similar levels of damage to the potato crop.

Furthermore, the field results only partially support trends observed in the laboratory susceptibility test. In the laboratory experiments, the risk of mortality after exposure to *M. brunneum* was at least slightly enhanced for the radish and mixed plant diets, and consequently we expected lower levels of damage to potatoes in plots with these cover crops. While this was indeed the case for potatoes planted after radish, the damage recorded after the cover crop mix was the highest among all treatments. These discrepancies between laboratory and field results may be explained by the limitations in space and time of the laboratory setting. Wireworms were kept in small containers in the laboratory, restricting the space for movement and potentially compromising natural behavior of the test insects. Avoidance of fungus-treated areas (Kabaluk and Ericsson 2007) is virtually impossible in these cups, and natural foraging behavior might be impeded. For example, it has been shown that wireworms produce different numbers and extent of burrows in the soil depending on the plant species present (Booth et al. 2020). Higher mobility may have enhanced the chance of contact with fungal spores in the soils of field plots, while our limited laboratory set-up did not allow observation of effects related to the movement of wireworms. Additionally, wireworms were feeding on growing plants in the field experiments, while they were offered seedlings or root cuttings during laboratory susceptibility tests. We cannot exclude that wireworms reacted differently to seedlings or root cuttings than to growing plants. Plant defense often changes during ontogenesis (Barton and Koricheva 2010) as well as when tissue damage occurs (Rasmann and Agrawal 2008). Lastly discrepancies may also be attributed to commonly occurring mixed species communities (Traugott et al. 2015) in the field, whereas only one species (*A. obscurus*) was tested in the laboratory. Nevertheless, overall it seems more probable that the reduced potato damage in the field originated from direct plant effects rather than from diet-mediated changes to wireworm susceptibility to *M. brunneum*.

Plant-mediated effects on wireworm damage have previously been tested for buckwheat, *Fagopyrum esculentum* (Moench) (Polygonaceae: Fagopyrum), (Bohorquez Ruiz et al. 2019), mustard, *Brassica juncea,* (L.) (Brassicales: Brassicaceae) (MacKenzie et al. 2010), marigold, *Tagetes* spp. (Asterales: Asteraceae), and calendula, *Calendula officinalis* (L.) (Asterales: Asteraceae) (Schepl and Paffrath 2007), but field results in these studies were mostly inconclusive. In general, host plants can directly affect insect performance through their nutrient content, with low-quality food leading to slower development, reduced lifespan, and lower fecundity (Barnett and Johnson 2013), as well as through secondary metabolites. Secondary metabolites are organic compounds that are strongly involved in the plant’s defense (Erb and Kliebenstein 2020). They can be toxic compounds (Sirikantaramas et al. 2008) or act in a regulating function for example by promoting the deposition of callose in the cell wall (Clay et al. 2009) or by inducing systemic resistance (Erb et al. 2015). In contrast to specialist herbivores that have evolved mechanisms to cope with these defense systems such as detoxification processes, generalist herbivores, like wireworms, may avoid these plants or dilute their effects by mixing their diet. In this way, they are able to reduce the consumption of toxins that occur in single plant species and gain further benefits from greater resource availability as well as the possibility to balance nutrient intake (Unsicker et al. 2008). Consequently, the physiological costs for generalists to overcome host defense may be higher when there is no choice and the host plant community is only composed of defended plants (Ali and Agrawal 2012, Thiel et al. 2020). This may have been the case in our field experiment, where the highest potato damage was recorded in plots with a mix of cover crops, whereas damage in clover and radish plots with EPF treatment was significantly lower.

Both clover and radish produce prominent secondary metabolites, namely isoflavonoids common in Leguminosae (Veitch 2013) and glucosinolates, defense compounds of the Brassicaceae (Essoh et al. 2020). Isoflavonoids have been described for their deterrent effects on generalist root herbivores (Johnson and Nielsen 2012). Glucosinolates come into contact with the plant enzyme myrosinase upon tissue damage, leading to the production of isothiocyanates and other biochemically active compounds (Halkier and Gershenzon 2006). Toxic and deterrent effects of these compounds have been widely documented for aboveground insect herbivores. Even though fewer studies have been performed for belowground herbivory (Sontowski et al. 2019), it is likely that they react similarly (Van Dam et al. 2009), especially as the content of glucosinolates is often higher in roots than in shoots (Tsunoda et al. 2018).

While the combination of EPF and oilseed radish resulted in a statistically significant reduction of wireworm damage in our study, the proportion of potatoes showing wireworm damage was still high (mean 17 ± 9%). Nevertheless, oilseed radish as a winter cover crop preceding potato may be a valuable and easy to apply tool in an integrated wireworm control strategy.

It is worth noting that the potato damage in bare fallow plots was not lower than that in plots with cover crops, contrary to what is sometimes suggested in agricultural guides (e.g., Funk 2018, Stammschröer 2018). This indicates that even over-winter periods of food deprivation do not substantially lower wireworm populations. Similar results have been obtained in other studies. In a 5-yr field trial comparing nontillage with winter cover crops to conventional tillage practices without cover crops and a thorough literature review, Furlan et al. (2021) concluded that interactions between wireworms and crop species cannot be generalized. Wireworm damage may be increased, decreased or invariable depending on the soil cover. Furlan et al. (2021) further found that diversified crop rotations including cover crops are favorable for soil biodiversity and thus may even encourage wireworm suppression.

In summary, there is strong evidence that wireworm foraging and feeding behavior can be influenced by the choice of the cover crop, and this might facilitate wireworm control. We were able to show that cover crops affected larval development and damage levels in potato. Even though the effects of cover crops on susceptibility to *Metarhizium* infection require further examination, perhaps including other plant species, it is worth recognizing forage plants as a potential factor when planning control setups. Some studies have already acknowledged this, for example in using trap crops (Landl and Glauninger 2013, Sharma et al. 2019) or attract-and-kill approaches using plants (Vernon et al. 2016) and forage search triggers (Brandl et al. 2017, La Forgia et al. 2021). Nevertheless, to successfully include selected forage crops in the plant protection system, it is necessary to further assess wireworm feeding behavior and to understand the underlying mechanisms of the effects of plant diet on wireworm survival.

## Supplementary Material

toac198_suppl_Supplementary_Figure_S1

## Data Availability

The datasets generated during and analyzed during the current study are available from the corresponding author on reasonable request.
