# Supplementary material for: Influence of Wireworm Diet on its Susceptibility to and Control With the Entomopathogenic Fungus Metarhizium brunneum (Hypocreales: Clavicipitaceae) in Laboratory and Field Settings
Source: J Econ Entomol. 2022 Dec 28;116(1):108–18. doi: 10.1093/jee/toac198 (PMC9912137; doi:10.1093/jee/toac198)
Supplement: toac198_suppl_Supplementary_Figure_S1 [file toac198_suppl_supplementary_figure_s1.docx]

Supplementary material


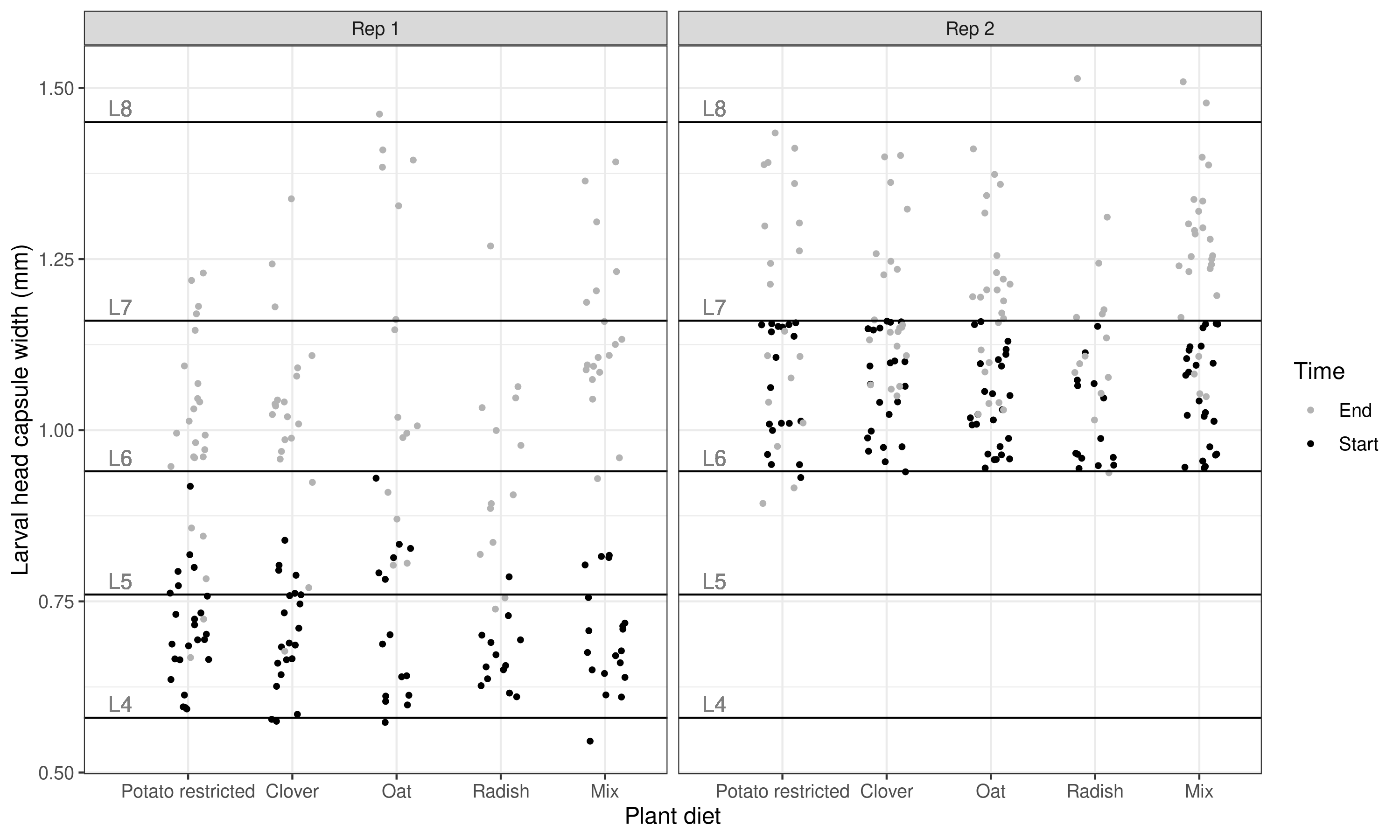


Figure S1. Head capsule widths of *Agriotes obscurus* larvae in the two replicates (Rep 1 & 2) of the laboratory experiment (*n* = 30 in each diet treatment). Measurements were taken at the beginning of the experiment (Start) and after 8 weeks of feeding on the specified plant diet (End). Lower boundaries for head capsule width in each larval instar according to Klausnitzer (1994) are indicated with horizontal lines.
